# Optimized nitrogen management enhances lodging resistance of rice and its morpho-anatomical, mechanical, and molecular mechanisms

**DOI:** 10.1038/s41598-019-56620-7

**Published:** 2019-12-30

**Authors:** Junfeng Pan, Junliang Zhao, Yanzhuo Liu, Nongrong Huang, Ka Tian, Farooq Shah, Kaiming Liang, Xuhua Zhong, Bin Liu

**Affiliations:** 1 Rice Research Institute, Guangdong Academy of Agricultural Sciences, Guangzhou, 510640 China; 2 Guangdong Key Laboratory of New Technology in Rice Breeding, Guangzhou, 510640 China; 3 Department of Agriculture, Abdul Wali Khan University, Mardan, Khyber Pakhtunkhwa Pakistan

**Keywords:** Transcription, Plant physiology

## Abstract

Increasing evidence shows that improved nitrogen management can enhance lodging resistance and lower internodes play a key role in the lodging resistance of rice. However, little is known about the cellular and molecular mechanisms underlying the enhanced lodging resistance under improved nitrogen management. In the present study, two rice varieties, with contrasting lodging resistance, were grown under optimized N management (OPT) and farmers’ fertilizer practices. Under OPT, the lower internodes of both cultivars were shorter but the upper internodes were longer, while both culm diameter and wall thickness of lower internodes were dramatically increased. Microscopic examination showed that the culm wall of lower internodes under OPT contained more sclerenchyma cells beneath epidermis and vascular bundle sheath. The genome-wide gene expression profiling revealed that transcription of genes encoding cell wall loosening factors was down-regulated while transcription of genes participating in lignin and starch synthesis was up-regulated under OPT, resulting in inhibition of longitudinal growth, promotion in transverse growth of lower internodes and enhancement of lodging resistance. This is the first comprehensive report on the morpho-anatomical, mechanical, and molecular mechanisms of lodging resistance of rice under optimized N management.

## Introduction

Maintaining high and stable grain yield of rice under limited supply of arable lands is an effective approach to meeting the ever-increasing food demand of growing global population. Nitrogen (N) fertilizer is essential for achieving desirable grain yield of rice^1^. But rice becomes excessively tall and thus susceptible to lodging under heavy and improper application of nitrogen fertilizer^2^. Besides, typhoons frequently occur every year in the southern provinces of China. On average, 2 to 3 typhoons land on the coast of Guangdong each year. When lodging occurs in the field, the grain yield of rice is lowered, grain quality is reduced, and mechanical harvest is hindered considerably^3,4^. Rice yield is usually decreased by 10–30% in South China^5^, and even up to 80% reduction in grain yield has been recorded under serious lodging conditions^3^. Thus, proper crop management (esp. nitrogen) should be developed to solve such problems^6,7^.

Previously, it has been found that lodging resistance is negatively related to the length of the lower internodes in rice^4,7,8^. Crop management, such as planting density, water management, timing and amount of nitrogen application, etc., dramatically affect the growth of lower internodes and the lodging resistance of rice^6^ and buckwheat^9^. In rice, the elongation of the first lower internode starts at the end of vegetative growth stage and overlaps with the panicle development^10^. The length of lower internodes is largely influenced by the cultivation practice, especially nitrogen fertilization. Heavy application of nitrogen fertilizer before internode elongation resulted in longer lower internodes and higher risk of lodging^7,11,12^. The plant height could be shortened and the lodging resistance could be improved by reducing the amount of nitrogen application^2^. However, the biomass production and grain yield are also reduced concomitantly under such circumstance^13^. A simultaneous increase in both grain yield and lodging resistance remains to be difficult in rice production, particularly in coastal areas such as South China.

To relieve the conflict between the lodging resistance and grain yield of rice, we developed a new technology in the past decade. It was named ‘three controls’ technology and possessed three major features, i.e., control of nitrogen fertilizer application, control of unproductive tillers, and control of pests and diseases. Compared to that of conventional farmers’ fertilizer practices of N application (FFP), fertilizer N input for ‘three controls’ technology is cut off by 50% during tillering, while N input after panicle initiation is more than doubled, resulting in a 20% reduction in total N input^14^. This technology is now very famous due to its superiority in lodging resistance in China^15–20^, especially in coastal areas where typhoon usually prevails during the rice-growing season. In addition, grain yield and nitrogen use efficiency are significantly improved as well. Farmers can typically save 20% nitrogen fertilizer and obtain 10% more grain yield compared to their conventional practice^14,21^. It was officially released in 2007 and was on the recommendation list of the Ministry of Agriculture of China in 2012 (http://www.moa.gov.cn/zwllm/tzgg/tfw/201202/t20120215_2481383.htm) as an optimized N management (OPT). It is now widely adopted in more than ten rice-growing provinces in China.

Despite its good practical importance, the mechanisms underlying the enhanced lodging resistance under OPT remains unclear. Although massive previous studies have found that lower internodes are responsible for lodging resistance of rice, little information is available on the changes in their anatomical and mechanical features. Besides, although it’s well known that the growth of lower internodes is largely influenced by nitrogen fertilization^7,11^, the genes involved in growth changes of the lower internodes under OPT are still largely unknown. In the present study, a comprehensive comparison between OPT and FFP was conducted at morpho-anatomical, mechanical and global gene expression levels. The objectives of this study were (1) to observe changes in morpho-anatomical features of lower internodes under two nitrogen managements, (2) to determine changes in mechanical properties of lower internodes in relation to lodging resistance of rice, and (3) to identify the genes responsible for enhancing lodging resistance in the lower internodes under OPT through global gene expression analysis.

## Results

### Effects of N management on length and plumpness of internodes

Among the five elongated internodes (≥1.0 cm) of rice, the three lowest were designated as lower internodes. The main difference between OPT and FFP was that N application at tillering stage was reduced by more than a half in contrast to FFP (Table 1). As shown in Tables 2 and S1, both individual length of three lower internodes and their total length under OPT were shorter than those under FFP for both varieties. The difference between FFP and OPT in the length of the first lower internode (I1) was non-significant, while the differences in the length of the second (I2) and third (I3) internodes and the total length of three internodes (I1 + I2 + I3) were statistically significant for both varieties. In contrast, the two upper internodes (I4 and I5) under OPT were both longer than those under FFP for both varieties. The change in length of three lower internodes in verification experiment had the same trend although it was non-significant (Table S1).

**Table 1:** Timing and rate of N, P and K fertilizer application under two nitrogen management practices in the field experiment.

Growth Stage	Farmers’ fertilizer practices (FFP)				Optimized N treatments (OPT)			
	Days after transplanting	N, amount (kg ha^−1^), rate (%)	K_2_O, amount (kg ha^−1^), rate (%)	P_2_O_5_, amount (kg ha^−1^), rate (%)	Days after transplanting	N, amount (kg N ha^−1^), rate (%)	K_2_O, amount (kg ha^−1^), rate (%)	P_2_O_5_, amount (kg ha^−1^), rate (%)
Basal dressing	−1	45, 30	67.5, 50	60, 100	−1	48, 40	67.5, 50	60, 100
Recovering and rooting	3	30, 20	—	—		—	—	—
Early tillering	7	45, 30	—	—		—	—	—
Mid-tillering		—	—	—	15	24, 20	—	—
Late tillering	20	30, 20	—	—		—	—	—
Panicle initiation	32	—	67.5, 50	—	32	36, 30	67.5, 50	—
Heading		—	—	—	70	12, 10	—	—
Total		150, 100	135, 100	60, 100	120, 100	135, 100	60, 100

**Table 2:** Internode length, plant height, gravity center height and grain yield under the two nitrogen fertilizer management practices.

Variety	Nitrogen management practice	Internode length(cm)						Plant height (cm)	Gravity center height (cm)
		I1	I2	I3	I1 + I2 + I3	I4	I5		
YJRZ	FFP	3.4 ± 0.1a*	8.1 ± 0.2a	16.9 ± 0.4a	28.4 ± 0.6a	18.4 ± 0.5a	36.8 ± 0.1a	104.0 ± 0.9a	43.9 ± 0.9a
	OPT	2.8 ± 0.2a	6.8 ± 0.5b	13.2 ± 0.6b	22.7 ± 0.9b	19.8 ± 0.6a	39.6 ± 0.6a	110.1 ± 3.3a	46.7 ± 0.8a
	%**	−18.3	−16.6	−22.1	−20.1	7.7	7.6	5.8	6.4
YZ889	FFP	3.6 ± 0.3a	8.7 ± 0.3a	17.8 ± 0.4a	30.1 ± 0.9a	16.3 ± 0.5b	34.6 ± 0.1b	102.9 ± 1.9a	45.5 ± 1.6a
	OPT	3.4 ± 0.2a	7.1 ± 0.5b	14.2 ± 0.5b	24.6 ± 1.1b	18.0 ± 0.7a	38.9 ± 0.4a	108.8 ± 2.1a	48.4 ± 1.4a
	%	−5.9	−18.7	−20.4	−18.2	10.7	12.3	5.7	6.5

Note: I1, I2, I3, I4 and I5 denote the 1^st^–5^th^ internodes being counted upwards from the base. *Mean ± standard error, values within a column followed by different letters are significantly different at the 0.05 probability level for a given variety. **Percent change of a certain trait under OPT is relative to that of FFP for a given variety.

The dry weight per unit length and dry weight per unit volume are indicators of internode plumpness. For both varieties, the dry weight of each lower internode under OPT was greater than that under FFP, respectively (Table 3). On average, the dry weight of the three lower internodes under OPT was increased by 13.3% for YJRZ and 21.9% for YZ889 compared with that under FFP. For both varieties, the dry weight per unit length of each lower internode under OPT was significantly greater than that under FFP, being increased averagely by 39.7% for YJRZ and 43.1% for YZ889. For YJRZ, the dry weight per unit volume of each lower internode under OPT was greater than that under FFP although the difference was not significant. For YZ889, the results were inconsistent.

**Table 3:** Culm dry weight, culm dry weight per unit length, and culm dry weight per unit volume of lower internodes under the two nitrogen fertilizer management practices.

Variety	Nitrogen management practices	Dry weight of internode (mg)			Dry weight per unit length of internode (mg cm^−1^)			Dry weight per unit volume of internode (mg cm^−3^)		
		I1	I2	I3	I1	I2	I3	I1	I2	I3
YJRZ	FFP	65.6 ± 2.9a*	93.2 ± 3.6a	126.1 ± 6.7a	19.5 ± 1.4b	11.9 ± 0.8b	7.6 ± 0.6b	131.6 ± 3.5a	115.3 ± 3.2a	101.6 ± 2.0b
	OPT	69.4 ± 4.5a	110.5 ± 7.6a	145.6 ± 5.9a	25.2 ± 0.7a	16.8 ± 0.5a	11.3 ± 0.5a	150.9 ± 9.8a	125.4 ± 6.5a	114.6 ± 1.5a
	%**	5.8	18.6	15.5	29.4	40.9	48.8	14.6	8.8	12.8
YZ889	FFP	55.9 ± 4.6a	103.8 ± 7.6a	126.3 ± 9.4b	15.7 ± 1.4b	12.6 ± 1.4b	7.1 ± 0.4b	223.7 ± 18.4a	205.5 ± 38.2a	123.8 ± 9.9a
	OPT	70.3 ± 6.5a	114.4 ± 10.2a	164.1 ± 8.0a	21.1 ± 1.2a	16.4 ± 0.4a	11.7 ± 0.2a	212.5 ± 16.3a	190.0 ± 9.7a	141.0 ± 6.3a
	%	25.8	10.1	29.9	34.4	29.5	65.5	−5.0	−7.6	13.9

Note: I1, I2 and I3 denote the 1^st^–3^rd^ internodes being counted upwards from the base. *Mean ± standard error, values within a column followed by different letters are significantly different at the 0.05 probability level for a given variety. **Percent change of a certain trait under OPT is relative to that of FFP for a given variety.

### Effect of N management on anatomical characteristics of lower internodes

For both varieties, the diameters of the three individual lower internodes under OPT were significantly bigger than those under FFP except for I1 of YJYZ (Table 4, *P* < 0.05). For both varieties, the culm wall thickness of each lower internode under OPT was significantly greater than that under FFP (*P* < 0.05). The culm volumes of three lower internodes under OPT were individually larger than those under FFP except for I1 of YJRZ, but all differences were not significant (*P* > 0.05). Overall, OPT could promote the transverse growth of lower internodes. The diameters and culm wall thickness of three lower internodes in verification experiment had the same trend (Table S2).

**Table 4:** Culm diameter, culm wall thickness, and internode volume of lower internode under the two nitrogen fertilizer management practices.

Variety	Nitrogen management practice	Culm diameter (mm)			Culm wall thickness (mm)			Culm volume (cm3)		
		I1	I2	I3	I1	I2	I3	I1	I2	I3
YJRZ	FFP	5.89 ± 0.14a*	5.45 ± 0.14b	4.85 ± 0.14b	0.93 ± 0.04b	0.67 ± 0.03b	0.54 ± 0.02b	0.49 ± 0.02a	0.81 ± 0.03a	1.23 ± 0.06a
	OPT	6.18 ± 0.18a	6.10 ± 0.19a	5.49 ± 0.15a	1.04 ± 0.02a	0.80 ± 0.02a	0.67 ± 0.03a	0.47 ± 0.05a	0.90 ± 0.10a	1.30 ± 0.03a
	%**	4.8	11.9	13.3	11.9	18.8	24.5	−3.8	11.7	5.7
YZ889	FFP	5.55 ± 0.10b	5.21 ± 0.12b	4.68 ± 0.16b	0.92 ± 0.01b	0.65 ± 0.02b	0.50 ± 0.01b	0.25 ± 0.03a	0.54 ± 0.05a	1.03 ± 0.03a
	OPT	5.93 ± 0.06a	5.84 ± 0.08a	5.56 ± 0.07a	1.03 ± 0.02a	0.79 ± 0.02a	0.61 ± 0.01a	0.33 ± 0.02a	0.61 ± 0.04a	1.16 ± 0.04a
	%	6.9	12.2	18.8	11.8	21.8	23.2	33.3	13.2	13.4

Note: I1, I2 and I3 denote the 1^st^–3^rd^ internodes being counted upwards from the base. *Mean ± standard error, values within a column followed by different letters are significantly different at the 0.05 probability level for a given variety. **Percent change of a certain trait under OPT is relative to that of FFP for a given variety.

**Figure 1:**
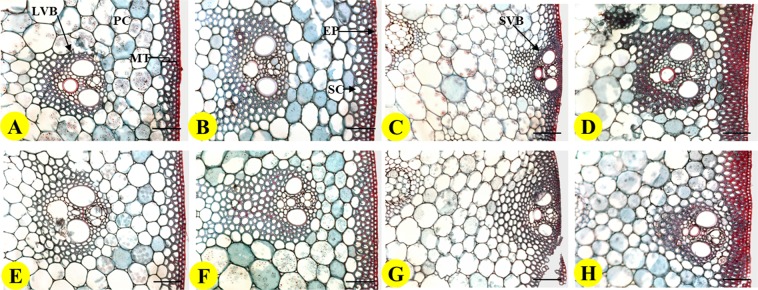
Comparisons of transverse sections in two lower internodes of two varieties under two nitrogen management practices. Transverse sections of the 1^st^ internode under FFP (**A**) and OPT (**B**) in YJRZ. Transverse sections of the 2^nd^ internode under FFP (**C**) and OPT (**D**) in YJRZ. Transverse sections of the 1^st^ internode under FFP (**E**) and OPT (**F**) in YZ889. Transverse sections of the 2^nd^ internode under FFP (**G**) and OPT (**H**) in YZ889. Scale bars indicate 50 μm. LVB, large vascular bundle; SVB: small vascular bundle; MT, mechanical tissue; PC, parenchymal cell; SC, sclerenchyma cell; EP, epidermis.

For both varieties, obvious differences between two treatments were also observed under microscope in the anatomical structures of two lower internodes (Fig. 1). Observation of the transverse sections of lower internodes revealed that the culm wall contained more sclerenchyma and parenchyma cells under OPT (Fig. 1). The cell walls of sclerenchyma cells below epidermis and vascular bundle sheath were thicker under OPT. On average, the thickness of mechanical tissue of I1 and I2 internodes under OPT treatment was 56.3% and 21.9% greater for YJRZ and YZ889 than those under FFP, respectively (Table 5). Except for I2 of YZ889, all other differences were significant. The sclerenchyma cell layers of both internodes were increased under OPT for both varieties. The difference in sclerenchyma cell layers was significant for YJRZ but non-significant for YZ889.

**Table 5:** Thickness of mechanical tissue and cell layer of sclerenchyma in lower internodes under the two nitrogen fertilizer management practices.

Variety	Nitrogen management practice	Thickness of mechanical tissue in internodes (μm)		Cell layer of sclerenchyma in mechanical tissue	
		I1	I2	I1	I2
YJRZ	FFP	45.1 ± 1.4b*	35.8 ± 5.1b	3.3 ± 0.1b	3.0 ± 0.3b
	OPT	61.2 ± 4.7a	63.3 ± 5.8a	4.0 ± 0.2a	4.8 ± 0.1a
	%**	35.6	77.0	21.8	59.7
YZ889	FFP	34.2 ± 2.1b	36.3 ± 3.6a	3.3 ± 0.2a	4.0 ± 0.0a
	OPT	45.5 ± 2.3a	40.2 ± 6.8a	3.6 ± 0.1a	4.9 ± 0.4a
	%	33.1	10.8	10.1	21.9

Note: I1 and I2 denote the 1^st^ and 2^nd^ internodes being counted upwards from the base. *Mean ± standard error, values within a column followed by different letters are significantly different at the 0.05 probability level for a given variety. **Percent change of a certain trait under OPT is relative to that of FFP for a given variety.

### Effect of N management on mechanical properties and lodging resistance of lower internodes

On average, the breaking resistance of I2 and I3 internodes under OPT treatment was 42.6% and 58.3% greater for YJRZ and YZ889 than those under FFP, respectively (Table 6). Except for I2 of YJRZ, all other differences were significant. The elastic modulus of both internodes was reduced under OPT for both varieties (Tables 6 and S3). The difference in elastic modulus was significant for YJRZ but non-significant for YZ889 (Table 6).

**Table 6:** Breaking resistance, elastic modulus, bending moment, bending stiffness, and lodging index of lower internodes under the two nitrogen fertilizer management practices.

Variety	Nitrogen management practice	Breaking resistance (N)		Elastic modulus (Gpa)		Bending moment (cm g)		Bending stiffness (×10^−3^ N m^2^)		Lodging index (cm g g^−1^)	
		I2	I3	I2	I3	I2	I3	I2	I3	I2	I3
YJRZ	FFP	9.1 ± 0.8a*	6.0 ± 0.6b	0.57 ± 0.09a	1.9 ± 0.14a	1155.3 ± 102.7a	760.0 ± 76.2b	17.1 ± 3.3a	32.3 ± 1.5a	151.9 ± 3.0a	204.1 ± 12.6a
	OPT	11.7 ± 0.8a	9.3 ± 0.3a	0.33 ± 0.04b	1.26 ± 0.05b	1490.3 ± 101.5a	1186.5 ± 32.0a	16.2 ± 3.3a	37.7 ± 2.0a	139.9 ± 11.5a	159.9 ± 7.5b
	%**	29.0	56.1	−41.8	−33.6	29.0	56.1	−5.3	16.5	−7.9	−21.7
YZ889	FFP	7.9 ± 0.6b	5.7 ± 0.5b	0.64 ± 0.04a	2.31 ± 0.32a	1005.0 ± 70.6b	728.8 ± 58.0b	17.7 ± 1.8a	36.2 ± 7.5a	168.1 ± 7.9a	185.7 ± 12.8a
	OPT	12.1 ± 0.4a	9.3 ± 0.3a	0.56 ± 0.11a	1.48 ± 0.16a	1548.4 ± 54.2a	1184.3 ± 36.6a	23.2 ± 4.5a	44.9 ± 3.7a	163.0 ± 6.3a	177.0 ± 3.3a
	%	54.1	62.5	−12.0	−36.2	54.1	62.5	30.5	24.2	−3.0	−4.7

Note: I2 and I3 denote the 2^nd^ the 3^rd^ internodes being counted upwards from the base, respectively. *Mean ± standard error, values within a column followed by different letters are significantly different at the 0.05 probability level for a given variety. **Percent change of a certain trait under OPT is relative to that of FFP for a given variety.

The bending moment under OPT was greater than that under FFP (Tables 6 and S3). Except for I2 of YJRZ, all differences were significant (Table 6). On average, the bending moment of the two internodes under OPT was increased by 42.6% for RJYZ and 58.3% for YZ889 (Table 6). Except for I2 of YJRZ, the bending stiffness was greater under OPT. However, all differences were non-significant. Lodging index, an indicator for lodging tendency or an opposite indicator of lodging resistance of rice, was reduced under OPT for both varieties (Table 6), although the difference was significant only for I3 of YJRZ. On average, the lodging index of I2 and I3 under OPT was 23% and 4% smaller for YJRZ and YZ889, respectively.

### Characteristics of gene expression profiling related to internode growth under two N management practices

**Figure 2:**
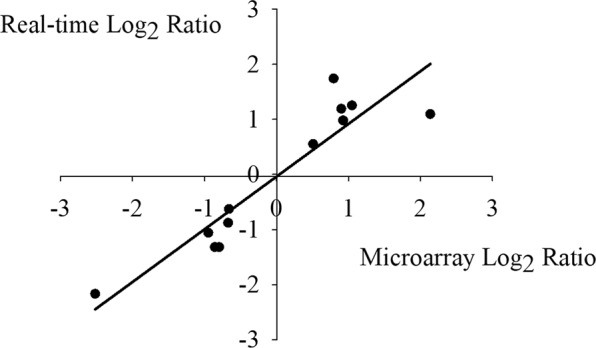
Comparison between the fold changes of the selected gene expression obtained by microarray and qRT-PCR. The data were collected from the expression of the selected 12 DEGs in microarray and Real-time PCR experiments. The fold changes in gene expression were transformed to log_2_ ratio. The microarray data log_2_ Ratio (X-axis) was plotted against the real-time RT-PCR log_2_ Ratio (Y-axis).

To understand the molecular regulating mechanism underlying the enhanced lodging resistance under OPT, genome-wide differential gene expression profiling of lower internodes from YJRZ was performed, using Agilent rice 44 K two-color microarrays. The pair-wise analysis showed that a total of 1378 differentially expressed genes (DEGs) were identified between OPT and FFP, with 665 genes being up-regulated and 713 genes being down-regulated. To validate the microarray data, 12 genes with up- or down-regulated or stable expression were randomly selected and their expression levels were detected by real-time qPCR with the same samples used in microarray assays. When the relative ratios between two treatments from either real-time qPCR or microarray readings were logarithmically converted and then plotted against each other, a good correlation between them could be established (R^2^ = 0.85, *P* < 0.01) (Fig. 2), proving the reliability of the microarray experiments in this study.

**Figure 3:**
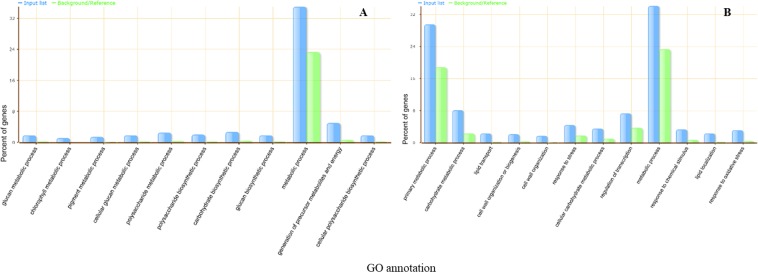
Over represented GO terms for DEGs of OPT compare to FP in biological process category. (**A**) GO terms of up-regulated genes. (**B**) GO term of down-regulated genes. GO terms are analyzed by AgriGO, a web-based tool, as described in the methods. The Y-axis is the percentage for the input DEGs in different GO terms calculated by the number of genes mapped to the GO terms divided by the number of all input genes. The same calculation was applied to the reference list to generate its percentage. The input lists are represented by blue color, while the reference lists are represented by green color. The X -axis is the definition of GO terms.

The up-regulated and down-regulated genes were subjected to GO analysis (Fig. 3). The most significant GO terms in up-regulated genes are mainly about carbohydrate and polysaccharide biosynthesis. On the other hand, the most significant GO terms in down-regulated genes include “cell wall organization and biogenesis” and “carbohydrate metabolic process”.

Three ADP-glucose pyrophosphorylase genes and 5 starch synthase genes, which are responsible for starch synthesis are among the up-regulated genes. Five genes encoding the key enzymes involved in lignin biosynthesis were also up-regulated under OPT (Table 7). On the other hand, many genes encoding cell wall loosening factors, including expansin, glycoside hydrolases and pectinesterase were included in the down-regulated genes under OPT (Table 7).

**Table 7:** Relative expression level of genes related to internode growth and lodging resistance in YJRZ under the two nitrogen management practices.

Gene ID	Log_2_(fold change)	Annotation
**ADP -glucose pyrophosphorylase**		
Os09g0298200	1.38	ADP-glucose pyrophosphorylase small subunit 1
Os05g0580000	1.462	ADP-glucose pyrophosphorylase large subunit 3
Os01g0633100	1.162	ADP-glucose pyrophosphorylase small subunit 2
**Lignin synthesis related genes**		
Os08g0157500	1.17	Caffeic acid 3-O-methyltransferase
Os07g0412100	1.10	Granule-bound starch synthase Ib
Os04g0518100	1.79	Phenylalanine ammonia-lyase
Os02g0627100	1.21	Phenylalanine ammonia-lyase
Os09g0419200	1.32	Phenylalanine ammonia-lyase
**Starch synthesis related genes**		
Os06g0160700	1.12	Starch synthase I
Os06g0133000	1.20	Starch synthase I
Os02g0744700	1.10	Starch synthase isoform
**Expansin**		
Os04g0228400	−2.44	Alpha-expansin OsEXPA
Os10g0556100	−1.94	Beta-expansin EXPB4
Os05g0477600	−1.81	Alpha-expansin OsEXPA4
Os02g0744200	−1.50	Alpha-expansin OsEXPA5
Os03g0336400	−1.42	Alpha-expansin OsEXPA4
Os07g0475400	−1.04	Expansin-like protein A
**Glycosyl hydrolase**		
Os06g0726200	−1.71	Endochitinase precursor
Os10g0160100	−2.02	Glycosyl hydrolases family 17
Os07g0420700	−1.80	Glycosyl hydrolase, family 31
Os01g0130400	−1.01	Glycosyl hydrolase, family 31
Os11g0673200	−1.39	Glycosyl hydrolase family 3 protein
Os05g0399400	−3.71	CHIT5 - Chitinase family protein
Os01g0860500	−1.11	Glycosyl hydrolase
Os04g0604300	−1.60	Xyloglucan endotransglucosylase
Os04g0631200	−1.60	Xyloglucan endotransglycosylase
Os06g0697000	−2.74	Xyloglucan endotransglycosylase
Os11g0539200	−2.11	Xyloglucan endotransglycosylase XET2
Os08g0237000	−1.12	XTH8_ORYSA Xyloglucan endotransglycosylase
Os06g0696400	−1.87	Xyloglycan endo-transglycosylase
Os03g0329500	−1.07	Endo-1,4-beta-glucanase
**pectinesterase**		
Os09g0571100	−1.06	Pectinesterase
Os01g0312500	−2.75	Pectinesterase
Os07g0675100	−1.27	Pectinesterase
Os08g0450100	−1.52	Pectinesterase

Note: RAP ID from Rice Annotation Project Database (RAP-DB): http://rapdb.dna.affrc.go.jp/. Data are presented as Log_2_ transform of the average of fold change from three replicates.

### Effect of N management on grain yield

The grain yield of YJRZ and YZ889 under OPT was 8.8% and 10.2% higher than that under FFP, respectively. Compared with FFP, the higher yield of YJRZ was mainly attributed to more spikelets per panicle under OPT, but for YZ889 the increase in yield could be explained by higher biomass and harvest index under OPT (Table 8). These results indicated that the interaction between genotype and nitrogen management might exist and this interaction should be considered in rice production.

**Table 8:** Yield, yield components, biomass and harvest index under the two nitrogen management practices.

Variety	Nitrogen management practices	Grain yield (g m^−2^)	No. of panicles (m^−2^)	Spikelets per panicle	Filled grain (%)	1,000-grain weight (g)	Biomass (g m^−2^)	Harvest index
YJRZ	FFP	724 ± 19a*	261 ± 10a	173 ± 3b	92.1 ± 0.7a	19.2 ± 0.1a	1219 ± 36a	0.56 ± 0.00a
	OPT	788 ± 18a	243 ± 8a	191 ± 3a	90.4 ± 0.6a	19.3 ± 0.3a	1221 ± 28a	0.57 ± 0.01a
	%**	8.8	−6.9	10.3	−1.8	0.5	0.2	1.2
YZ889	FFP	773 ± 19b	237 ± 11a	159 ± 3a	82.2 ± 1.8a	26.5 ± 0.1a	1261 ± 68b	0.56 ± 0.01b
	OPT	852 ± 19a	251 ± 10a	171 ± 7a	84.3 ± 1.8a	26.6 ± 0.1a	1431 ± 22a	0.58 ± 0.00a
	%	10.2	6.1	7.5	2.5	0.3	13.4	3.0

Note: *Mean ± standard error, values of a same variety within a column followed by different letters are significantly different at the 0.05 probability level. **Percent change of a certain trait under OPT is relative to that of FFP in a same variety.

## Discussion

### Lower internodes being shortened but being broadened and thickened under OPT

Under OPT, N fertilizer was applied at panicle initiation stage, i.e. 32 days after transplanting, which was the late elongation stage of second lower internode (Table 1). Prior to this, the amount of nitrogen fertilizer applied under OPT was only half of that under FFP. At such lower dosage of nitrogen fertilizer, the nitrogen content of plants under OPT would be also low^22^. Thus, the longitudinal growth of the first and second lower internodes would be inhibited but their transverse growth would be promoted under OPT. Besides, a 15-day-long gap between the two topdressings under OPT might have also widened the difference in their growth pattern. During the elongation of the third lower internodes, the nitrogen content of plants under FFP might be still relatively higher due to enormous accumulation of nitrogen during the preceding period. Meanwhile, a time lag might exist between the nitrogen application and growth response, so the third lower internodes under OPT were still relatively shorter but thicker. Previous studies showed that lower internodes became shorter under low nitrogen condition^7,11^. A limited supply of nitrogen fertilizer in the mid-season shortens the lower internodes and enhances the lodging resistance of rice^23^. In contrary, heavy basal dressing of nitrogen can result in excessive elongation of lower internodes^6,11^. Our recent study showed that crop N concentration at jointing stage had no direct effect on lower internode length. Nitrogen fertilization indirectly affects lower internode length through changing leaf area index and light environment at the base of canopy^24^. It has been reported that GA induces both cell division and cell elongation and thus GA can influence several traits such as plant height, the length of internodes, lodging resistance, plant architecture, and biomass production etc^25^. When the nitrogen application was reduced, *in vivo* gibberellins synthesis was decreased and the shoot and root elongation was inhibited^26^. Although we did not measure changes in GA content, our findings are not contradictory to the supposition that GA plays an important regulatory role in internode development. Further studies are needed to better understand the mechanism of lower internodes elongation under OPT.

In contrast, most farmers using FFP usually apply fertilizer-N 2~3 times within 15 days after transplanting and almost all fertilizer-N is used as basal or tillering fertilizers^5^. This practice can certainly lead to not only lower nitrogen use efficiency, but also huge number of unproductive tillers, which would result in dense crop canopy and low light intensity at the base of the canopy^14,27^. Under such dense and shading conditions, the synthesis of cellulose and lignin in both sclerenchyma and parenchyma cells is reduced consequently, resulting in poor culm strength and higher lodging risks^28^.

### Mechanical strength of lower internodes being remarkably improved under OPT

The breaking resistance of lower internodes, together with its two components of elastic modulus and bending stiffness, are usually used as major parameters to evaluate the lodging resistance of rice plants. In this study, the breaking resistance and bending stiffness of both varieties were greater but the elastic modulus was smaller under OPT (Table 6). Previous study also showed that the elastic modulus and bending stiffness had a negative correlation^29^. The elastic modulus measures the rebounding force after occurrence of temporary bending, while bending stiffness directly measures the resistance capacity to external force before occurrence of permanent bending. Once permanent bending forms and lodging occurs, it cannot be restored by rebounding force. This suggests that the elastic modulus might play a limited role only when a slight and temporary bending occurs while bending stiffness plays a big role in breaking resistance. The increase in breaking resistance under OPT might be mainly caused by the enhanced bending stiffness.

The three lower internodes had both greater internode weight and dry weight per unit length of internode under OPT (Table 3). The previous studies reported that if more non-structural carbohydrate accumulated in the stems after heading, their lodging resistance could be significantly enhanced^6,30,31^. Moreover, it has been also reported that the dry weight of internode and bending stiffness were positively and significantly correlated^11,12^. It was found that the bending stiffness was enhanced by increased dry-matter density, and was positively and significantly correlated with glucose, xylose and lignin content of rice plants^32^. A recent study also indicated that there were significant positive correlations between basal culm strength and dry weight and cellulose and lignin proportions, and high-yielding rice populations with greater culm strength had greater carbohydrates accumulation^12^. Our result is consistent with these reports. The higher bending moment and bending stiffness under OPT brought about a significantly greater breaking resistance in both varieties (Table 6). The above results indicate that three mechanical properties related to lodging (breaking resistance, bending moment, and bending stiffness of lower internodes) were all improved remarkably under OPT.

### Lodging resistance, plant height and grain yield being moderately enhanced under OPT

The lodging index is an important parameter of lodging tendency in rice, and rice plants with lower lodging index are more tolerant to lodging than those with higher lodging index. In this study, the lodging index was lower under OPT than under FFP for both varieties due to a higher breaking resistance under OPT (Table 6), indicating that the lodging resistance of rice plant was improved under OPT treatment and this improvement was independent to varietal types. In comparison trials in farmer’s fields, lodging frequently occurred under FFP and seldom took place under OPT^15–17,20^.

Plant height is closely associated with lodging resistance, biomass and grain yield in rice. Previously, high plant was usually thought to reduce the lodging resistance of rice^7^. In this study, the plant height and gravity center height were both increased, but the lodging resistance was also enhanced under OPT (Tables 2 and 6). Single stem weight (biomass per m2/panicles per m2) was greater under OPT. Although the weight of lower internodes under OPT was greater, the weight of other parts could be also greater, and hence the center of gravity of the plant was not necessarily lower. In fact, the center of gravity under OPT was even higher than that under FFP, although not significant (Table 2). It should be pointed out that the increase in lodging resistance under OPT resulted mainly from improved breaking resistance (Table 6) due to greater culm diameter and culm wall thickness (Table 4), rather than lower plant height or center of gravity of the plant. The positive effect of improved breaking resistance on lodging resistance might have surpassed the negative effect of higher plant height or plant gravity center under OPT. It has been reported that rice cultivars with similar plant height differed greatly in the susceptibility to lodging^29,33^. In contrast, good lodging resistance was reported in rice varieties with tall plant height^30^. These results and facts indicate that lower internodes play more important role than plant height in the lodging resistance of rice, and the increase in lodging resistance under OPT was resulted mainly from improved breaking resistance (Table 6) due to greater culm diameter and culm wall thickness of lower internodes (Table 4), rather than lower plant height or center of gravity of the plant. Thus, it is possible that the plant height of rice can be moderately increased with no penalty of lodging resistance if the growth of lower internodes is properly controlled under a better N management like OPT (Tables 2 and 5).

Although the rate of N application under OPT condition was reduced by 20% with a same application frequency (Table 1), the yields of both varieties under OPT were increased by 8.8–10.2% in comparison to those under FFP. The higher yield was mainly attributed to the increase in panicle size, biomass, and harvest index under OPT (Table 8). Before this study, we had studied the effect of OPT on the growth and nitrogen utilization of rice and had published a relevant paper^21,22^. Under FFP, all nitrogen fertilizer was applied before panicle initiation. This led to the excessive growth of tillers and many of them became unproductive and died gradually after panicle initiation^22^. Under OPT, although only half of nitrogen was applied before panicle initiation, the quantity of tillers produced was still big enough to meet the requirement for panicle number^21,22^. Compared with that under FFP, the maximum number of tillers was reduced by 5.9% and the number of effective tillers increased by 12.3% under OPT^22^. Moreover, the amount of nitrogen uptake under OPT was lower before panicle initiation, but it was higher in the middle and late periods of growth, and the SPAD value of functional leaves under OPT were higher from two weeks before heading to maturity^22^. Finally, OPT showed advantages in sink size (no. of panicles per square meter × spikelets per panicle), dry matter (biomass) accumulation and harvest index (Table 8). As filled grain rate and grain weight were usually constant, the grain yield was thus higher under OPT (Table 8). Overall, the grain yield can be increased without penalty of lodging resistance under OPT.

### Transcription of genes related to elongation, cell wall enforcement and starch synthesis in lower internodes being differentially regulated under OPT

Many genes responsible for lignin synthesis and starch accumulation were found to be up-regulated under OPT. Lignin are major biopolymer components of cell wall in plant, and lignin synthesis is related to mechanical strength of culm^13,28,34,35^. It has been reported that the loss-function of a rice cinnamyl-alcohol dehydrogenase gene, which participates in lignin synthesis, led to dramatic reduction in mechanical strength^36^. Caffeic acid 3-O-methyltransferase is a key enzyme participating in lignin synthesis. Lodging-resistant genotype of wheat had higher expression level of this enzyme gene compared to the susceptible genotype^37^. In this study, we found that the gene encoding caffeic acid 3- O-methyltransferase (*Os08g0157500*) had higher expression level under OPT. Many other lignin synthesis genes were also identified to display similar expression difference between the two treatments (Table 7). Higher expression level of genes participating in lignin synthesis could lead to higher lignin content and higher mechanical strength of lower internodes. These changes were also confirmed by our microscopic observation (Fig. 1).

Starch accumulation in lower internodes was supposed to be partly responsible for the increase in lodging resistance of rice^38^. A study on lodging-related QTL in rice demonstrated that the increase in both dry weight of internodes and lodging resistance were caused by the accumulation of non-structural carbohydrates (starch in particular) in lower internodes^30^. The non-structural carbohydrates contributed to lodging resistance by increasing the physical strength of culms and leaf sheaths^31,38^. Our results showed that increase in the culm weight, culm diameter and culm wall thickness of lower internodes under OPT was caused by the enhanced expression level of genes participating in the starch synthesis pathways. In another word, up-regulation of those genes under OPT can promote the transverse growth of lower internodes.

In contrast, many genes encoding cell wall loosening factors, including expansin, glycosyl hydrolase, and pectinesterase, were found to have lower expression level under OPT in this study. Expansins are one of the major classes of hydroxyproline-rich glycoproteins that can promote cell wall loosening and extension and are encoded by a super family of genes^39^. Even though the biochemical properties of expansins have not yet been fully characterized, they have been proved to play important roles in plant growth^40^. In deep water rice, the expression of expansin genes was shown to be positively correlated with internode elongation^41^. In transgenic plants of rice, the protein level of *OsEXP4* (a rice expansin) was also found to be positively correlated with plant growth^42^. However, a recent study^43^ found that a novel extensin-like (*OsEXTL*) gene can significantly reduce cell elongation in stem internodes, and reduce plant heights by 7–10%. Besides, the *OsEXTL*-transgenic plants had remarkably thickened secondary cell walls with higher cellulose levels, resulting in significantly increased mechanical strength in the mature transgenic plants^43^. The above contrary results show that different expansin genes from the same gene family may have opposite effects on internode development and lodging resistance in rice.

Plant glycoside hydrolase, another cell wall loosening factor, is involved in the polysaccharide degradation of cell wall, which is an important metabolic process during cell division, expansion, and differentiation^44^. Families 9, 16, 17, and 31 of glycoside hydrolase are supposed to be involved in cell wall assembly^45^. In this study, genes for glycoside hydrolase families had lower expression level under OPT. Xyloglucan endotransglycosylase (*XET*) belongs to the family 16 of glycoside hydrolase and it (*XET*) internally cleaves xyloglucan polymers and ligates the newly generated reducing ends to other xyloglucan chains. Because xyloglucan mediates the cross-linking of cellulose microfibrils in the plant cell wall, XET has a big role in cell wall plasticity and cell elongation^46^. A rice XET of *OsXTH8* (*Os08g0237000*) has been characterized and was found to be involved in rice internode elongation. Its expression level was positively correlated with the height of rice mutants^47^. In this study, 4 genes encoding XET including *OsXTH8*, showed lower expression level under OPT. Endo-b-1,4-D-glucanase is a cell wall-loosening enzyme which participates in the modification of the cell wall. In this study, a gene encoding rice membrane-bound endo-1, 4-b-D-glucanase, *OsGLU1* (*Os03g0329500*), was found to have lower expression level under OPT. *OsGLU1* has previously been proven to affect the internode elongation of rice^48^.

Pectin is one of the structural polysaccharides in the middle lamella and primary cell walls of higher plants. Pectinases, also one of cell wall loosening factors, are functionally to degrade pectin and may also play a major role in plant cell elongation^49^. In this study, 4 genes encoding pectinesterase were shown to have lower expression level under OPT.

Overall, cell wall loosening factors, including expansin, glycoside hydrolases and pectinesterase, all had lower gene expression under OPT, resulting in lowered cell expansion and division and reduced the length of three lower internodes in rice.

### Comprehensive understanding of improved lodging resistance under OPT

**Figure 4:**
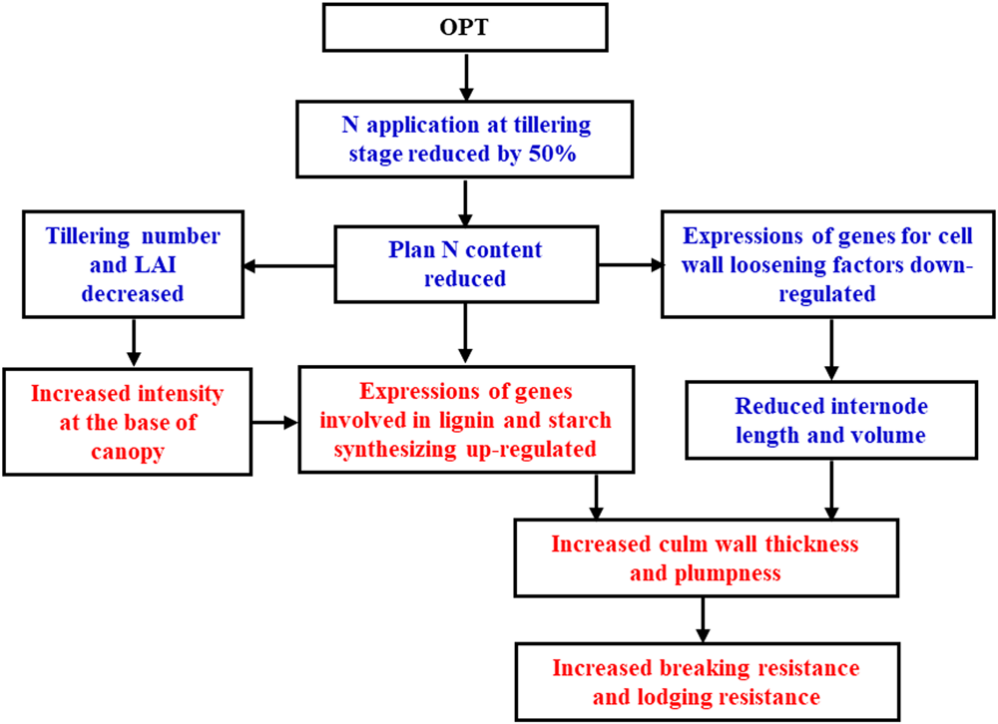
Illustration of lodging resistance of rice being enhanced under OPT. Red color indicates that traits or the expressions of relevant genes were increased or up-regulated under OPT; blue indicates that traits or the expressions of relevant genes were decreased or down-regulated.

For a given rice variety, the enhancement in lodging resistance can be fundamentally attributed to the changes in global gene expression under OPT. As elucidated above, the transcript levels of genes for cell wall loosening factors was decreased under OPT, which could reduce the cell elongation and lead to reduction in internode length (Tables 2 and S1). The transcription of genes participating in lignin and starch synthesis was up-regulated, which could increase the content of carbohydrates and structural substances of cell, and finally contribute to the increased culm wall thickness (Tables 4 and S2) and matter accumulation (Table 3). Besides, some major isoenzyme genes were differentially expressed under two nitrogen regimes, imply that they are potential targets of metabolic engineering for improvement of lodging resistance in rice. The differential expression of above-mentioned genes would bring about some conducive morpho-anatomical changes below: (1) reduced internode length and volume, (2) increased culm wall thickness, and (3) increased internode plumpness. Based on those beneficial morpho-anatomical changes, the mechanical properties of lower internodes were therefore improved, which accounted directly for the enhanced lodging resistance (Fig. 4). This study is the first comprehensive report on the morpho-anatomical, mechanical, and molecular mechanisms of lodging resistance of rice under optimized nitrogen management. However, a complete picture of the systematic basis of the lodging trait and its contribution to grain yield under OPT awaits further analyses.

## Conclusion

Compared with those under FFP, the total length of three lower internodes was reduced by 18–21% while their culm diameter, culm wall thickness, plumpness, and breaking resistance were significantly increased under OPT. Lodging index was decreased under OPT in both varieties with different lodging resistance. The genes related to the cell wall loosening factors were down-regulated while the genes participating in lignin and starch synthesis were up-regulated, leading to shorter and thicker internodes and enhanced lodging resistance under OPT.

## Materials and Methods

### Plant materials

Two rice (*Oryza sativa* L.) varieties with similar growth duration (having 2 days difference in heading date), Yinjingruanzhan (YJRZ, an inbred variety with weak lodging resistance) and Yueza 889 (YZ889, a hybrid variety with medium lodging resistance), were chosen for this study. Both varieties are Guangdong’s major varieties and have been most widely cultivated during the last decades in Guangdong province.

### Field experimental design and growth conditions

A field experiment was conducted in the early cropping season (March to July) of 2012 at Dafeng Experimental Farm in Guangzhou (23°10′N, 113°20′E, altitude of 41.0 m), Guangdong province, China. The experiment was laid out in a split-block design with two N managements as the main plot and two varieties as the subplot treatments with four replicates. There were 16 subplots in total and the size of each subplot was 15 m^2^.

Thirty-day-old seedlings were transplanted on April 7, 2012 at a spacing of 0.2 m × 0.2 m with 2 (for YZ889) or 4 (for YJRZ) seedlings per hill. We used fewer seedlings per hill for hybrid rice according to farmers practice^50,51^. Hybrid rice has much stronger tillering ability and hybrid rice seeds are much more expensive than inbred rice. Two N management treatments, farmer’s fertilizer management (FFP) and optimized fertilizer management (OPT), were established in the main plots. The amount, ratio and timing of fertilizer N application in FFP were determined based on our survey of farmer’s fertilizer practice in Guangdong province, China (Table 1). Under FFP, all nitrogen is basal dressed or top dressed during tillering stage prior to panicle initiation. Under OPT, the total nitrogen input was reduced by 20% compared to that of FFP, and the ratio and timing of split application were all adjusted. Most of the nitrogen application was delayed as up to 40% of nitrogen being applied after panicle initiation. N fertilizer was applied in the form of urea. Potassium (135 kg K ha^−1^ as KCl) was split into two applications with half being basal dressed and half being top dressed at 30 days after transplanting. Phosphorus (60 kg P ha^−1^as single super-phosphate) was all applied as basal fertilizer (Table 1). All basal fertilizers were incorporated into fields one day before transplanting. Pests, diseases, and weeds were intensively controlled to avoid yield loss.

Means of daily minimum temperature, maximum temperature, relative humidity, and sunshine hours were 23.5 °C, 30.6 °C, 84.9% and 4.0 in 2012 early season, respectively. The total rainfall and from transplanting to maturity was 884.2 mm (Fig. S1).

### Verification experiment

Two rice (*Oryza sativa* L.) varieties with similar growth duration (5 days difference in heading date), Yinjingruanzhan (YJRZ, an inbred variety with weak lodging resistance) and Huanghuazhan (HHZ, an inbred variety with medium lodging resistance), were chosen for verification study.

The field verification experiment was conducted in the early cropping season (March to July) of 2012 at Baiyun Experimental Farm (35 km away from Dafeng Experimental Farm) in Guangzhou (23°17′N, 113°23′E). The experiment was laid out in a split-block design with two N managements as the main plot and two varieties as the subplot treatments and with four replicates. There were 16 subplots in total and the size of each subplot was 20 m^2^.

Thirty-day-old seedlings were transplanted on April 4, 2012 at a spacing of 0.2 m × 0.2 m with 4 seedlings per hill. Two N management treatments, farmer’s fertilizer management (FFP) and optimized fertilizer management (OPT), were established in the main plots. The amount of N application under FFP and OPT was 165 kg N ha^−1^ and 150 kg N ha^−1^, respectively. The split ratio and timing of fertilizer N application in FFP and OPT were same with the experiment in Dafeng Experimental Farm (Table 1). The amount, ratio and timing of fertilizer P and K application and field management in FFP and OPT were same with the experiment in Dafeng Experimental Farm. Lodging resistance traits were measured at 15 days after heading for both varieties, and the measuring method was same with the experiment in Dafeng Experimental Farm.

### Measurement of internode morphological traits

At 15 days after heading, ten main culms were sampled from each subplot to measure the plant height (distance between the plant base and the tip of the panicle), gravity center height (distance between the geometric center of gravity and the base of stem), panicle length, and the length of five individual internodes (I1, I2, I3, I4 and I5). The first internode was defined as the first elongated lower internode (≥1.0 cm) near the ground, and other internodes were labeled upwards sequentially. There were five elongated internodes for both tested varieties. Internodes were divided in the middle of nodes by one sided blade, and the fresh weight of panicle and five individual internodes was separately measured. Culm diameter and culm wall thickness were both measured in the middle of internode using a caliper (Digital Caliper 0–100 mm, Lugong Inc, China). Dry weight of individual internodes was measured after oven-drying at 80 °C to constant weight. Dry weight per unit length of internode (mg cm^−1^) = the dry weight of internode/the length of internode.

### Measurement of mechanical parameters

Three lower internodes from ten main culms were sampled for each replicate to determine the average breaking resistance at 15 days after heading according to the method described by Li *et al*.^7^, using a portable lodging resistance detector (YYD-1, Tuopu instruments co., ltd, China). The fixed distance between the two supporting points was 5 cm, the internodes were placed horizontally on the supporting points and the force required for breaking the internode at the middle point was recorded. The minimum force of internode breaking was considered as the breaking resistance. If the length of the internode was less than 5 cm, the breaking resistance was not measured. Mechanical parameters were then calculated according to the methods described by Islam *et al*.^4^ and Niklas^52^. Elastic modulus was measured in bending degree by horizontally suspending each axis, loading it at its mid length (L/2) with a force F, and measuring the resulting vertical deflection δ (in unit of cm) at L/2. The value of elastic modulus (E) was computed using the formula: E = F × L3/(48 × δ × *I*), where L denotes the effective length of internode (i.e. the distance between the two vertical braces supporting the axis). Values for elastic modulus were computed and recorded only for axes that elastically restored their original curvature after the bending load was removed. *I* is the internode section moment of area (in unit of mm3): *I* = π × a4/4 × [1 − (1 − t/a)^4^], where, a is the radius of internode, t is the culm wall thickness. Bending stiffness (N m^2^) = Section modulus × the internode section moment of area. Lodging index (LI) = Bending moment /Breaking resistance. Bending moment (g cm) = the distance from the basal of internode to tip of panicle × the fresh weight of this section. Culm dry weight per unit length of internode (mg cm^−1^) = the dry weight of internode/internodes length. Culm dry weight per unit volume of internode (mg cm^−3^) = the dry weight of internode/culm volume. Culm volume (cm3) = π × L × (2 × a × t − t2).

### Microscopical observation of lower internode transverse section

The first and second lower internodes from four main culms were sampled for each replicate at 15 days after heading and were fixed immediately in formalin-acetic acid alcohol (FAA) for no less than 24 h. They were then dehydrated by gradually passing through a series of ethanol gradient, cleaned in xylene and embedded in paraffin wax according to the method described by Wang^53^. A 10 μm-thick transverse section in the middle of lower internodes was obtained using a rotary microtome, stained with double dyeing reagents of safranine and fast green, and observed and photographed under an optical microscope (LEICADM 4000B, Germany). Safranine and fast green are known to dye lignified sclerenchyma tissue (stained red) and cellulose-rich parenchyma tissue (stained blue) separately. Adobe Photoshop CS6 software was used to Image analysis. The measurement parameters were set according to the pixel points represented by the scale, and the built-in measurement function of software was used to measure the thickness of different tissues in sliced pictures. Perpendicular to the epidermis, the distance from the surface of the epidermis to the outer wall of the innermost sclerenchyma was measured in mechanical tissue of internodes, and 3–5 observations were measured for each sample.

### Total RNA extraction

About 0.1 g (fresh weight) first lower internode of YJRZ was sampled at about 30 days after transplanting and the growth stage of sampled internodes was determined by examining stems. Total RNA was extracted with Trizol reagent (Invitrogen, Carlsbad, CA, USA) and purified with Nucleo Spin RNA Clean-up reagent (MACHEREY-NAGEL, Germany). Both quality and quantity of RNA were assessed by formaldehyde denaturing agarose gel electrophoresis and spectrophotometry (Nanodrop-1000, Thermo-Fisher, USA), respectively.

### Microarray analysis

Agilent 44k rice oligo nucleotide microarray (No: 015241, Agilent Technologies, USA) which contains 45,018 features/microarray and - 40,000 transcripts were used and processed. RNA amplification and cDNA labeling were conducted using cRNA Amplification and Labeling Kit (CapitalBio, China), according to the manufacturer’s protocol. cDNAs from first internode of rice plants of FFP were labeled with Cy3 dye. cDNAs from samples of OPT were labeled with Cy5 dye and paired with FFP for microarray assay. Microarray slides were hybridized for 16 hours at 65 °C with hybridization buffer containing two paired labeled samples in final concentration of 5X Denhardt, 3X SSC and 0.2% SDS. After hybridization, the slides were subjected to four-step stringency washes (2X SSC + 0.2% SDS, twice at 42 °C for 4 min; 0.2X SSC, twice at 42 °C for 4 min). Microarrays were scanned using Axon 4000B scanner and analyzed by Genepix Pro 6.0 (Axon Instruments, USA). Three biological replicates were used for each pair of OPT and FFP. The raw data from microarrays was filtered by Genepix Pro 6.0. Data were further normalized using the Lowess method in Acuity 4.0 (Axon Instruments, USA). DEGs were identified using the combined criteria of two-fold change and a cut-off of *p*-values (≤0.05) with mean of three biological replicates. Gene Ontology (GO) analysis was carried out using the Singular Enrichment Analysis tool offered by agriGO (http://bioinfo.cau.edu.cn/agriGO/index.php)^54^. The original microarray data set of this study has been deposited in NCBI’s Gene Expression Omnibus (http://www.ncbi.nih.gov/geo/) under GEO series number GSE103063.

### Validation of microarray data with real-time quantitative PCR (qRT-PCR)

A total of 12 genes representing three types of expression data in microarray: up regulation, down regulation and no change, were selected for qRT-PCR validation. The same RNA used in microarray assays was used in qRT-PCR validation. Total RNA was reverse transcribed into cDNA using PrimeScript reverse transcriptase (Takara, Japan). The real-time PCR was conducted using Ex-Taq SybrGreen PCR Mix (Takara, Japan) in Biorad CFX-96 real-time PCR machine (Biorad, USA). All samples were processed in three biological replicates.

### Determination of yield and yield components

Grain yields were determined at maturity by taking 5 m2 plant samples at the center of each plot. Grains were separated from the rachis, filled and unfilled grains were separated, and the total weight of filled grains was determined. Filled grains were dried in an oven at 70 °C to a stable weight, and grain yield was calculated at 14% moisture content. Plant samples were taken for the determination of yield components from 12 hills (0.48 m^2^) adjacent to the harvest area. The panicles m^−2^, spikelets per panicle, filled grain, 1000-grain weight, and harvest index were all calculated. Total dry weight was calculated by summing the total dry matter of straw, rachis, filled and unfilled spikelets. Harvest index was calculated as the percentage of grain yield to the total biomass.

### Data analysis

Statistical analysis was performed by Statistix (Version 9.0, Statistix Institute). Analyses of variance (ANOVA) were made to determine the significance of difference between two nitrogen managements for a given variety. All of the figures were constructed and the regression analysis were performed using SigmaPlot 12.5.

## Supplementary information

Supplementary Information.
