# Supplementary material for: Optimized nitrogen management enhances lodging resistance of rice and its morpho-anatomical, mechanical, and molecular mechanisms
Source: Sci Rep. 2019 Dec 30;9:20274. doi: 10.1038/s41598-019-56620-7 (PMC6937289; doi:10.1038/s41598-019-56620-7)
Supplement: Supplementary file 1 — Supplementary Information. [file 41598_2019_56620_MOESM1_ESM.docx]

Supplementary Information (SREP-19-10759C)

**Optimized nitrogen management enhances lodging resistance of rice and its morpho-anatomical, mechanical, and molecular mechanisms**

**Junfeng Pan**^1,2,^**^†^** , **Junliang Zhao**^1,2,^**^†^**, **Yanzhuo Liu**^1,2^**, Nongrong Huang**^1,2^**, Ka Tian**^1,2^**, Farooq Shah**^3^**, Kaiming Liang**^1,2,^ **Xuhua Zhong**^1,2,#^**, & Bin Liu**^1,2,#^

^1^ Rice Research Institute, Guangdong Academy of Agricultural Sciences, Guangzhou 510640, China

^2^ Guangdong Key Laboratory of New Technology in Rice Breeding, Guangzhou 510640, China

^3^Department of Agriculture, Abdul Wali Khan University, Mardan, Khyber Pakhtunkhwa, Pakistan

**^†^** These authors contributed equally to this work.

^#^ Corresponding authors,

Dr. Xuhua Zhong, Dr. Bin Liu

^1^ Rice Research Institute, Guangdong Academy of Agricultural Sciences

^2^ Guangdong Key Laboratory of New Technology in Rice Breeding

Guangzhou 510640,

China

Tel: (+86 20)8756 9473 (Dr Zhong)

(+86 20)85162404 (Dr Liu)

Email: xzhong8@163.com (Dr Zhong)

lbgz1009@163.com (Dr Liu)

Journal for submission: *Scientific Reports*

Fig. S1 Means of daily maximum and minimum temperatures (A), daily relative humidity (B), daily rainfall (C), and daily sunshine hours (D) at the experimental field from transplanting to maturity in Guangzhou, Guangdong Province, China in 2012.

Table S1. Internode length, plant height, gravity center height and grain yield under the two nitrogen fertilizer management practices.

| Variety | Nitrogen management practice | Internode length(cm) | | | | | Plant height(cm) |  | Gravity center height(cm) |
| --- | --- | --- | --- | --- | --- | --- | --- | --- | --- |
|  |  |  |  |  |  |  |  |  |  |
|  |  | I1 | I2 | I3 | I1+I2+I3 | I4 |  |  |  |
| YJRZ | FFP | 3.1 a* | 7.7 a | 15.5 a | 26.3 a | 18.8 a | 104.1 a |  | 43.3 a |
|  | OPT | 2.8 a | 6.2 a | 12.7 a | 21.6 a | 18.8 a | 104.1 a |  | 43.8 a |
|  | %** | -11.0 | -20.0 | -18.3 | -17.9 | 0.1 | 0.0 |  | 1.2 |
| HHZ | FFP | 3.1 a | 7.5 a | 11.7 a | 22.2 a | 17.0 a | 97.6 a |  | 43.6 a |
|  | OPT | 2.7 a | 6.4 a | 11.6 a | 20.7 a | 17.4 a | 98.8 a |  | 43.6 a |
|  | % | -13.5 | -14.1 | -0.7 | -7.0 | 2.3 | 1.3 |  | 0.1 |

Note: I1, I2, I3, and I4 denote the 1^st^ - 4^th^ internodes being counted upwards from the base. *Values within a column followed by different letters are significantly different at the 0.05 probability level for a given variety. **Percent change of a certain trait under OPT is relative to that of FFP for a given variety.

Table S2. Culm diameter, culm wall thickness, and internode volume of lower internode under the two nitrogen fertilizer management practices.

| Variety | Nitrogen management practice | Culm diameter | | |  | Culm wall thickness | | |  | Culm volume | | |
| --- | --- | --- | --- | --- | --- | --- | --- | --- | --- | --- | --- | --- |
|  |  | (mm) | | |  | (mm) | | |  | (cm^3^) | | |
|  |  | I1 | I2 | I3 |  | I1 | I2 | I3 |  | I1 | I2 | I3 |
| YJRZ | FFP | 5.9 a* | 5.7 a | 4.9 a |  | 1.0 a | 0.7 a | 0.6 a |  | 0.5 a | 0.9 a | 1.2 a |
|  | OPT | 6.1 b | 6.0 a | 5.3 a |  | 1.1 a | 0.8 a | 0.6 a |  | 0.5 a | 0.8 a | 1.1 a |
|  | %** | 3.5 | 5.8 | 7.7 |  | 8.2 | 7.1 | 9.8 |  | -1.3 | -8.8 | -3.5 |
| HHZ | FFP | 5.5 a | 5.2 b | 4.5 b |  | 1.0 a | 0.7 b | 0.6 a |  | 0.4 a | 0.8 a | 0.8 b |
|  | OPT | 5.9 a | 5.7 a | 5.1 a |  | 1.0 a | 0.8 a | 0.6 a |  | 0.4 a | 0.8 a | 1.0 a |
|  | % | 7.2 | 9.1 | 11.7 |  | 5.7 | 8.4 | 9.6 |  | -1.4 | 0.9 | 21.3 |

Note: I1-I3 denote the 1^st^, 2^nd^ and 3^rd^ internodes being counted upwards from the base. *Values within a column followed by different letters are significantly different at the 0.05 probability level for a given variety. ** Percent change of a certain trait under OPT is relative to that of FFP for a given variety.

Table S3. Breaking resistance, elastic modulus, bending moment, and bending stiffness of lower internodes under the two nitrogen fertilizer management practices.

| Variety | Nitrogen  Management  practice | Breaking  resistance (N) | |  | Elastic modulus (Gpa) | |  | Bending  moment (cm g) | |  | Bending  stiffness | |
| --- | --- | --- | --- | --- | --- | --- | --- | --- | --- | --- | --- | --- |
|  |  |  |  |  |  |  |  |  |  |  | (×10^-3^N m^2^) | |
|  |  | I2 | I3 |  | I2 | I3 |  | I2 | I3 |  | I2 | I3 |
| YJRZ | FFP | 10.0 a* | 7.6 b |  | 0.6 a | 8.7 a |  | 1278.2 a | 968.1 b |  | 19.0 a | 165.0 a |
|  | OPT | 11.9 a | 10.0 a |  | 0.4 a | 5.7 a |  | 1517.6 a | 1272.3 a |  | 16.7 a | 144.2 a |
|  | %** | 18.7 | 31.4 |  | -31.9 | -35.0 |  | 18.7 | 31.4 |  | -12.2 | -12.6 |
| HHZ | FFP | 12.8 b | 10.3 b |  | 1.0 a | 6.8 a |  | 1633.2 b | 1317.1 b |  | 25.1 a | 94.6 a |
|  | OPT | 15.0 a | 11.7 a |  | 0.6 b | 6.0 a |  | 1911.5 a | 1496.9 a |  | 22.2 a | 125.1 a |
|  | % | 17.0 | 13.7 |  | -37.3 | -12.2 |  | 17.0 | 13.7 |  | -11.7 | 32.3 |

Note: I2 and I3 denote the 2^nd^ and the 3^rd^ internodes being counted upwards from the base, respectively. *Values within a column followed by different letters are significantly different at the 0.05 probability level for a given variety. ** Percent change of a certain trait under OPT is relative to that of FFP for a given variety.
